# Diet inequality prevails among consumers interested and knowledgeable in nutrition

**DOI:** 10.3402/fnr.v59.27601

**Published:** 2015-11-23

**Authors:** Andreas Håkansson, Håkan S. Andersson, Yvonne Granfeldt

**Affiliations:** 1 Food and Meal Science, School of Education and Environment, Kristianstad University, Kristianstad, Sweden; 2 Linnaeus University Centre for Biomaterials Chemistry, Linnaeus University, Kalmar, Sweden; 3 Department of Chemistry and Biomedical Sciences, Linnaeus University, Kalmar, Sweden; 4 Department of Food Technology, Engineering and Nutrition, Lund University, Lund, Sweden

**Keywords:** diet cost, diet quality, energy density, micronutrient density, diet inequality, nutritional education

## Abstract

**Background:**

Previous studies have demonstrated a correlation between diet cost and adherence to nutritional recommendations among consumers in general. This has adverse effects on diet and health inequality. It could be hypothesized that consumers knowledgeable in nutrition escape this correlation.

**Objective:**

Investigate whether the previously observed relationship between diet cost and nutritional quality prevails among consumers with an above-average interest in and knowledge of nutrition.

**Design:**

Full open diet registrations of 330 students taking a basic university-level course in nutrition over a total of 780 days.

**Results:**

The consumers with the highest daily average diet cost differ from the lowest cost quartile: The diets had higher micronutrient density, more fruits and vegetables, and lower energy density. The highest cost daily diet quartile had a significantly higher energy adjusted intake of the micronutrients that were on average consumed below the recommendation (vitamin D, folate, and iron for women). On the other hand, alcohol intake was significantly higher among the high diet cost group. The highest diet cost respondents consumed more fish, meat, coffee, and spreads, whereas the lowest diet cost respondents had a higher consumption of cereals, bread, jam, sausage, and milk.

**Conclusions:**

Dietary differences prevail even in the above-average interested and knowledgeable group. The respondents did not use their higher level of knowledge to break this commonly observed relationship. This suggests that an increased minimum level of knowledge in nutrition may not by itself eliminate dietary inequality.

A positive relationship between the compliance of diets to official nutritional recommendations (henceforth: diet quality) and diet cost among the general population is well established through previous studies: A higher diet cost is associated with lower energy density and/or higher intake of key nutrients (1–3), higher consumption of fruit and vegetables (1, 4–6), higher consumption of fish (1, 5), and higher nutritional density (1, 2, 5, 7). Furthermore, studies have shown better diet quality among high socioeconomic status (SES) households (8, 9) and a higher risk for diet-related illnesses among low SES individuals in Sweden (10–12) and elsewhere (13–16). Foods that are more nutrient rich, more energy poor, and generally classified as ‘healthy’ are often more expensive than alternatives (17–21). It has been argued that diet and health inequality is mediated by the higher cost of nutritious foods (22, 23). This is also supported by the finding that the nutrients that are consumed below recommendations in Sweden are the most costly to obtain for rational and well-informed individuals (24).

In understanding how to alleviate diet and health inequality, it is of interest to clarify whether the relationship between diet quality and diet cost is possible to avoid for a consumer with special habits, knowledge, and background (6). Intervention studies have shown that it is possible to increase nutritional compliance without increasing cost (25–27), and it is known that education has a beneficial effect on diet quality (4, 7, 28). Thus, it could be hypothesized that consumers well versed in nutrition would be able to escape from the diet cost–diet quality correlation. If this hypothesis holds, it would imply that increased educational efforts in nutrition could alleviate diet inequality.

The overall objective of this study is to better understand whether above-average interested and knowledgeable consumers are an exception to the general population and have diet quality independent of diet cost. The specific questions are as follows: 1) Does the previously established relationship between diet cost and diet quality prevail in the knowledgeable sample? 2) Does this sample display the same differences in dietary patterns as previous studies report for the general population? 3) Does this group also show a relationship between under-consumed nutrients and the cost of individual nutrients?

## Materials and methods

### Survey, sample, and methodology

Students actively participating in university-level basic nutrition courses were used as a sample of above-average interested and knowledgeable consumers. The study is based on full open diet recording (food recording) from 397 students enrolled in courses in nutrition at Linnaeus University (‘Food, Nutrition, and Health’) or Kristianstad University (‘Basic Nutrition’ and ‘Nutrition I’) 2013–2014. Students conducted the registrations approximately two thirds into a first cycle level course corresponding to 5 weeks’ full-time studies. They were given an introduction to nutrition assessments in general and diet recordings in particular before participating. They were then instructed to record and report all foods and masses thereof (preferably by direct weighing) consumed during at least 2 days using commercial dietetic software (Dietist Net, Kostdata, Bromma, Sweden) at least one weekday (Monday–Thursday) and one weekend day (Friday–Sunday). One-fifth of the students (the Kristianstad cohort) also estimated their physical activity level (PAL) during the period. The rest of the students were assumed to have the average PAL of the Kristianstad cohort (=1.6, corresponding to sedentary work and a somewhat active lifestyle outside of work). A 95% Goldberg cutoff (29) was used to exclude individuals reporting unreasonable intake for any day. After this filtering, 330 respondents reporting in total 780 days remained, and these were used for all further analysis.

Reported amounts of the different foods were translated to intake of different macro- and micronutrients using primarily the national Swedish nutrition database (30). For food items not included in the national database (0.038% of the items), the United States Department of Agriculture (USDA) database (31) and the producers’ own Dabas database (32) were used. Each of the foods in the database were assigned to 1 of the 25 food groups (see Table 3), designed so as to correspond to the grouping of a national Swedish survey (33).

Since these surveys were part of an examination at Swedish universities, they are publicly accessible documents according to Swedish regulations. All surveys were made anonymous by the professor responsible for each course to ensure anonymity of the respondents. Great care was taken to ensure that none of the reported results could be traced to individual respondents: All data reported are given as means or medians of no fewer than 30 individuals.

### Food price data

Price data for the 2,088 food items in the nutritional database were estimated from online supermarkets. Prices were obtained during the first half of 2014. The average daily diet cost of each of the respondents was estimated assuming all foods were prepared and consumed in the home. Similar price estimation has been used in previous similar studies (1, 6, 7), and the method was suggested in a recent comparison (34).

### Energy-adjusted cost and intake

Cost and intake of foods and nutrients were normalized so as to correspond to an intake of 10 MJ per day. Adjusting to the same energy intake makes comparison between individuals with varying nutrient requirements easier. Furthermore, energy-adjusted cost increases comparability to previous studies.

### Linear programming for shadow prices

Linear programming offers a method of investigating the cost of general nutritional recommendations as experienced by a rational and knowledgeable consumer (35–38). Using shadow price analysis, it is also possible to estimate how costly a set of nutrition recommendations is for a cost-minimizing consumer (39). Shadow prices were obtained from a previous study (24) [based on Swedish prices, dietary habits from a national survey (33), and Nordic nutritional recommendations, NNR (40)] and were compared to the actual average intake in order to investigate whether there is a relationship between which nutrients are costly and which are consumed below recommendations.

### Energy and micronutrient density

The diet energy density for each individual was calculated by dividing the total energy intake (as calculated from the survey and nutrition database) by the total mass of reported intake. The micronutrient density was calculated as the sum of the ratio of reported to recommended intake of 18 nutrients (see Table 2) according to the Nordic nutrition recommendations (40), normalized to an energy intake of 10 MJ per day:

$$\text{ND}=\frac{10\text{MJ}}{TE}\sum_{j=i}^{J}\frac{m_{j}}{M_{j}}$$

where *m*_*j*_ is the reported average daily intake and *M*_*j*_ the recommended daily intake of nutrient *j* (*j=*1 … 18). The measure is similar to Drewnowski's ‘nutrient rich food index’ (41) adjusted to Nordic recommendations.

### Data analysis and statistics

The respondents were divided in quartiles based on their energy-adjusted average daily diet cost. Student's *t*-tests were used to investigate if mean intake of the top and bottom quartile differed significantly for the variables following a normal distribution (as judged by a Kolmogorov–Smirnov test). A non-parametric Mann–Whitney *U*-test was used to test for significant differences between medians of non-normally distributed variables. The type of test used is indicated in the result. Results were judged as statistically significant if *p<*0.05. All statistical tests were used as implemented in MATLAB 2013b (MathWorks, Natick, MA).

## Results

### Diet quality and macronutrients

Measures of diet quality over the quartiles can be seen in Table 1. Significant differences can be seen between quartiles: intake of fruits and vegetables is significantly higher, and energy density is significantly lower in the top quartile. Individuals with a higher diet cost also have higher micronutrient density. For alcohol, on the other hand, the higher diet cost quartiles have a higher average consumption although still within the recommended interval. For proteins, the highest cost quartile has an intake above the recommended maximum corresponding to an excessive 2 g/d of proteins. A high diet cost is associated with consuming less carbohydrates and fat and more proteins. It could be noted that all group mean intakes fall within the range of recommended intake for the macronutrients (see Table 1) except for sodium, for which the intake is excessive for all quartiles, and for the previously mentioned protein for the high cost quartile.

**Table 1:** Comparison of demographic variables with diet characteristics and nutritional quality divided in quartiles (*Q*1–*Q*4) of average diet cost

	Type	*Q*1 (*n=*83)	*Q*2 (*n=*82)	*Q*3 (*n=*82)	*Q*4 (*n=*83)	Total (*n=*330)	*p*	Rec^c^
Energy-adjusted diet cost (SEK/d/10 MJ)	Median (IQR)	77 (9.5)	93 (7.2)	110 (8.3)	130 (25)	99 (30)	<0.001§	
Diet cost (SEK/d)	Median (IQR)	72 (29)	83 (24)	85 (32)	110 (38)	87 (36)	<0.001§	
Age (years)	Median (IQR)	24 (6)	25 (7.8)	25 (8.0)	25 (7.8)	25 (8.0)	0.032§	
BMI (kg/m^2^)	Median (IQR)	22 (3.2)	22 (3.0)	23 (4.1)	23 (3.5)	22 (3.4)	0.810§	
Gender (% men)	Mean (STD)	23 (42)	13 (35)	15 (33)	12 (34)	16 (36)	0.100†	
Surveyed number of days	Median (IQR)	2 (1.0)	2 (1.0)	2 (1.0)	2 (1.0)	2 (1.0)	0.920§	
Number of different foods consumed per day	Median (IQR)	45 (25)	50 (29)	47 (24)	48 (27)	48 (27)	0.420§	
Total energy per day (MJ/d)	Median (IQR)	9.5 (3.8)	9.0 (2.8)	7.9 (2.9)	8.3 (2.2)	8.8 (3.0)	0.011§	
Energy% carbohydrates	Median (IQR)	49 (9.2)	50 (11)	47 (8.3)	44 (13)	47 (11)	<0.001§	45–60
Energy%, fat	Median (IQR)	35 (8.4)	35 (10)	35 (8.6)	34 (9.3)	35 (9.1)	0.039§	0
Energy%, protein	Median (IQR)	16 (4.5)	18 (4.5)	18 (5.5)	20 (8.0)	18 (5.1)	<0.001§	10–20
Energy%, *cis* polyunsaturated fatty acids	Median (IQR)	5.4 (3.0)	5.3 (3.1)	5.2 (3.2)	5.4 (3.5)	5.3 (3.2)	0.520§	5–10
Dietary fibers (g/d)	Median (IQR)	25 (1.7)	27 (2.1)	25 (2.0)	24 (2.8)	26 (2.1)	0.360§	25–35
Greens^a^ (g/d)	Median (IQR)	500 (390)	590 (410)	500 (370)	520 (500)	530 (410)	0.041§	>500
Alcohol^b^ (g/d/10 MJ)	Median (IQR)	0 (0.0)	0 (0.0)	0 (6.6)	0 (18)	0 (4.7)	<0.001§	<17
Na (g/d/10 MJ)	Mean (STD)	3.0 (1.1)	3.2 (1.3)	3.1 (0.98)	3.3 (1.2)	3.2 (1.1)	0.140†	<2,300
Energy density (kJ/g)	Mean (STD)	4.9 (1.2)	4.2 (1.3)	4.2 (1.1)	3.8 (1.0)	4.3 (1.2)	<0.001†	
Micronutrient density, ND	Median (IQR)	16 (6.7)	17 (6.1)	18 (6.0)	20 (8.2)	17 (6.7)	<0.001§	

*p*-Value for (two-sided) difference between *Q*1 and *Q*4 calculated as a *t*-test for the difference between means (†) or Mann–Whitney *U*-test for difference between medians (§), depending on whether the variable is normally distributed according to a Kolmogorov–Smirnov normality test.

a Sum of fruits, berries, fruit juices, vegetables, and root vegetables excluding potatoes

b calculated based on the recommendation of <5% of the energy from alcohol (40);

c recommended (Rec) intake for adults according to NNR (40).

### Individual nutrients

Some differences can be seen in individual nutrients (Table 2). (The table shows energy-adjusted intake for the female respondents due to differences in recommendations and the low percentage of male respondents.) The high diet cost quartile has a significantly higher energy-adjusted intake of several vitamins (vitamins B6, B12, C, D, and E, folate, niacin, and riboflavin) and minerals (iron, iodine, potassium, magnesium, phosphorous, selenium, and zinc). The differences are not unimportant; three nutrients are on average consumed below recommendations (vitamin D, folate, and iron), for all there are significant differences in intake between the highest and lowest diet cost quartiles.

**Table 2:** Intake of vitamins and minerals for the female respondents, *N*=278, divided in quartiles (*Q*1–*Q*4) based on average daily diet cost

Nutrient		*Q*1 (*n=*69)	*Q*2 (*n=*70)	*Q*3 (*n=*69)	*Q*4 (*n=*70)	Total (*n=*278)	Total of RDI^a^ (%)	*p*	National average^b^
Vitamin A (RE/10 MJ)	Median (IQR)	830 (711)	950 (530)	850 (540)	940 (900)	900 (600)	130	0.530§	860±360
Vitamin B6 (mg/10 MJ)	Median (IQR)	2.3 (1.1)	2.7 (1.3)	2.7 (1.0)	2.9 (1.2)	2.7 (1.2)	210	0.002§	2.3±0.8
Vitamin B12 (µg/10 MJ)	Median (IQR)	5.8 (4.8)	6.1 (6.0)	6.7 (5.6)	7.9 (6.1)	6.6 (5.8)	330	0.003§	5.3±2.2
Vitamin C (mg/10 MJ)	Median (IQR)	140 (80)	170 (130)	160 (120)	170 (170)	160 (110)	230	0.004§	110±57
Vitamin D (µg/10 MJ)	Median (IQR)	5.3 (7.9)	6.8 (11)	7.9 (11)	7.5 (10)	6.6 (10)	82	0.012§	6.7±3.4
Vitamin E (mg/10 MJ)	Median (IQR)	14 (6.5)	16 (9.0)	15 (7.0)	17 (10)	15 (8.1)	200	0.008§	14±7.0
Folate (µg/10 MJ)	Median (IQR)	340 (130)	420 (180)	410 (240)	420 (190)	390 (200)	98	<0.001§	300±77
Niacin (NE/10 MJ)	Median (IQR)	39 (14)	40 (16)	42 (16)	47 (17)	41 (16)	270	<0.001§	23±7.3
Riboflavin (mg/10 MJ)	Median (IQR)	1.8 (0.51)	1.9 (0.53)	2.0 (0.63)	2.0 (0.74)	1.9 (0.63)	140	0.017§	1.8±0.5
Thiamin (mg/10 MJ)	Median (IQR)	1.4 (0.51)	1.5 (0.66)	1.6 (0.65)	1.5 (0.63)	1.5 (0.64)	130	0.350§	1.4±0.3
Ca (g/10 MJ)	Mean (STD)	0.92 (0.4)	1.0 (0.43)	1.0 (0.38)	0.98 (0.47)	0.99 (0.43)	120	0.400†	1,100±300
Fe (mg/10 MJ)	Median (IQR)	12 (3.6)	13 (4.5)	14 (4.7)	15 (4.5)	14 (4.7)	94	<0.001§	11.9±3.1
I (µg/10 MJ)	Mean (std)	170 (68)	190 (110)	210 (100)	210 (100)	200 (99)	125	0.036†	NA
K (g/10 MJ)	Median (IQR)	3.6 (0.76)	3.9 (1.4)	4.0 (1.1)	4.0 (1.2)	3.9	120	<0.001§	3,600±880
Mg (mg/10 MJ)	Median (IQR)	380 (93)	430 (130)	440 (130)	420 (140)	420 (120)	140	<0.001§	380±86
P (g/10 MJ)	Mean (STD)	1.6 (0.31)	1.7 (0.38)	1.8 (0.43)	1.8 (0.48)	1.7 (0.41)	270	0.003†	1,600±260
Se (µg/10 MJ)	Median (IQR)	53 (24)	61 (41)	63 (39)	72 (48)	63 (37)	130	<0.001§	50±17
Zn (mg/10 MJ)	Mean (STD)	12 (3.3)	12 (3.9)	13 (3.3)	14 (5.4)	13 (4.1)	180	0.014	12±2.7

NE, niacin equivalents: 1 niacin equivalent=1 mg niacin=60 mg tryptophan; RE, retinol equivalents: 1 retinol equivalent=1 µg retinol=12 µg β-carotene. *p*-Value for (two-sided) difference between *Q*1 and *Q*4 calculated as a *t*-test for the difference between averages (†) or Mann–Whitney *U*-test for difference between medians (§), depending on whether the variable is normally distributed according to a Kolmogorov–Smirnov normality test. NA, not available; IQR, interquartile range; STD, standard deviation.

a Recommended daily intake (RDI) for a woman 18–31 years of age with an intake of 10 MJ/d (40);

b Swedish national average (33) for women 18–30 years per 10 MJ.

### Food patterns

The differences in diet quality and nutrient intake can be related to differences in what foods are consumed by individuals with low and high diet costs. Table 3 shows the average intake of 25 food groups by quartiles and compared to a larger general Swedish survey from 2011 (33). The highest diet cost quartile consumes more root vegetables, vegetables and fruits, and berries. They also consume more expensive but protein-rich foods such as meat and fish. In terms of beverages, the higher quartile consumes more caffeinated (coffee and tea) and alcoholic beverages. Consumption of spreads and oils is also higher in the high cost quartile.

**Table 3:** Mass of food (g) consumed from the different categories per 10 MJ of energy intake divided in quartiles (*Q*1–*Q*4)

Food group	*Q*1^a^ (*n=*83)	*Q*2^a^ (*n=*82)	*Q*3^a^ (*n=*82)	*Q*4^a^ (*n=*83)	Total^a^ (*n=*330)	*p*	National^b^ 2011
Spreads, fats	22 (48)	21 (50)	24 (83)	64 (160)	28 (68)	<0.001§	15
Cheese	18 (32)	32 (41)	18 (39)	15 (30)	20 (37)	0.068§	33
Milk	290 (240)	270 (290)	280 (270)	210 (250)	260 (270)	0.022§	330
Bread	86 (81)	67 (75)	57 (57)	55 (75)	65 (74)	0.024§	120
Potatoes	65 (140)	54 (130)	74 (130)	50 (130)	62 (130)	0.31§	130
Root vegetables	0 (48)	32 (67)	25 (68)	20 (78)	22 (67)	0.041§	29
Vegetables	180 (140)	250 (250)	260 (260)	250 (290)	240 (221)	<0.001§	210
Fruits, berries	170 (160)	260 (280)	260 (230)	240 (290)	230 (250)	0.002§	170
Fruit juices	0 (120)	0 (89)	0 (25)	0 (0)	0 (72)	<0.001§	76
Pasta	0 (27)	0 (7.4)	0 (3.0)	0 (2.2)	0 (8)	0.054§	36
Meat	150 (160)	160 (180)	180 (190)	220 (230)	170 (200)	0.009§	120
Eggs	40 (82)	46 (80)	38 (70)	56 (110)	40 (79)	0.39§	19
Fish	28 (80)	69 (95)	68 (100)	95 (150)	69 (120)	<0.001§	55
Sausage	0 (17)	0 (1.0)	0 (1.5)	0 (2.3)	0 (2.3)	0.013§	29
Biscuits	7.9 (43)	9.7 (49)	11 (41)	0 (38)	3.5 (41)	0.30§	40
Ice cream	0 (1.2)	0 (4.3)	0 (2.0)	0 (2.4)	0 (3.0)	0.061§	11
Pastries	0 (4.1)	0 (8.3)	0 (2.2)	0 (7.2)	0 (9.9)	0.054§	23
Jam	0 (3.8)	0 (1.0)	0 (2.3)	0 (5.1)	0 (4.2)	<0.001§	13
Soft drinks	93 (660)	0 (830)	5.7 (580)	0 (640)	1.7 (660)	0.87§	150
Sweets	6.1 (28)	5.1 (23)	11 (27)	0 (19)	5.7 (24)	0.41§	17
Sugar	0 (4.3)	0 (5.9)	0 (67)	0 (3.2)	0 (8.1)	0.884§	2.6
Coffee and tea	99 (260)	210 (450)	230 (460)	250 (390)	190 (420)	0.003§	620
Alcoholic beverages	0 (0.0)	0 (0.0)	0 (96)	0 (180)	0 (61)	<0.001§	200
Nuts, seeds, and legumes	16 (41)	15 (49)	18 (41)	13 (34)	15 (40)	0.56§	11
Cereals	210 (208)	140 (130)	170 (190)	120 (120)	150 (180)	0.021§	100
Total	2,100 (640)	2,500 (1,200)	2,400 (670)	2,600 (1,000)	2,400 (990)	<0.001§	2,600

*p*-Value for (two-sided) difference between *Q*1 and *Q*4 calculated as a *t*-test for the difference between averages (†) or Mann–Whitney *U*-test for difference between medians (§), depending on whether the variable is normally distributed according to a Kolmogorov–Smirnov normality test.

a Reported as median (interquartile range);

b National per-day average per 10 MJ (33).

The lowest quartiles, on the other hand, consume more starchy foods – bread and cereals – together with jam, sausage, dairy products, and fruit juices. The high diet cost group consumes a larger total mass of food per day, largely due to a high intake of energy-poor fruits and vegetables.

### Demographic differences between groups

There are no significant differences in body mass index (BMI) or gender (Table 1) over diet cost quartiles. However, the high diet cost quartile has a higher average age. The age of respondents ranges from 18 to 61 years; the difference in average age is small, but the percentage of older students is significantly higher in *Q*4, where 12% of the respondents are over 40 years old (compared to 6% in *Q*1).

### Comparison to shadow prices

**Fig. 1:**
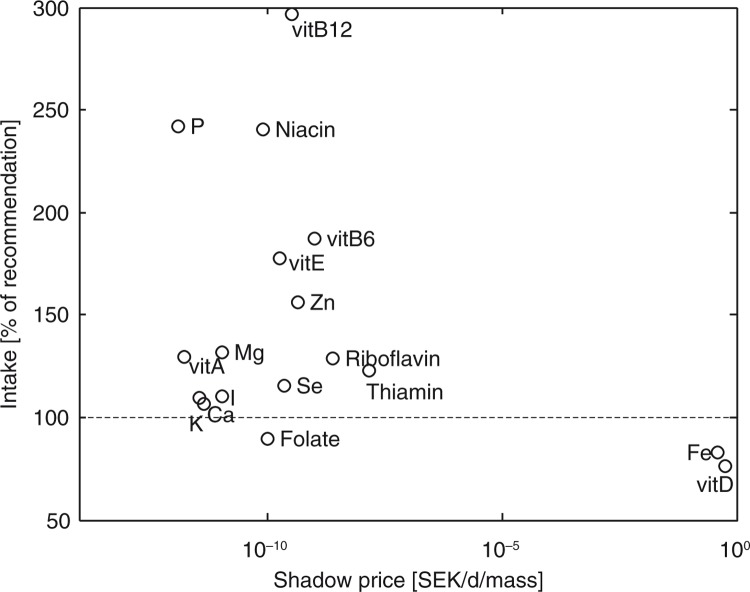
The price of each nutritional constraint – the shadow price – compared to the daily intake (averaged over all women respondents) as percentage of recommendation (RDI) for women aged 18–30 years.

Figure 1 compares the average intake of the 18 micronutrients in Table 2 to the implicit cost of each nutrient recommendation in the form of a shadow price, see (24). The two nutrients with highest shadow prices (vitamin D and iron) show insufficient intake as compared to the NNR, (40) in the sample. Furthermore, all nutrients with intake above 150% of recommended intake have low shadow prices. In summary, the nutrients most costly to obtain are consumed below recommendations by the knowledgeable consumers in the sample, and the nutrients consumed in excess have a very low cost.

## Discussion

### Difference in diet quality

This study sets out to investigate whether the previously reported differences in nutritional quality over energy-adjusted diet cost can be found even within a group of nutritionally knowledgeable consumers and uses university-level students as a sample of this population. As seen in the results, this sample does not differ much from the general population as described by previous studies. The previously reported higher diet energy density for consumers with lower diet cost (1–3, 7) is found for this group as well. The same is true for higher micronutrient density (1, 4, 6, 7, 42) and the previously found differences in intake of fish (1, 5) and fruit and vegetables (1, 5, 6).

Due to differences in methodology and large within-individual variations, it is difficult to determine whether the effect is larger or smaller than for general consumers. However, it is noteworthy that the relative difference between average energy density of the highest and lowest quartiles of this study is larger than the relative difference between that reported by Andrieau et al. (2) for a representative French sample and also higher than the relative difference between highest and lowest quantiles of Ryden and Hagfors for Swedish children (1). This indicates that the differences within the knowledgeable group are not necessarily smaller than those in a more general sample.

An interesting exception to the trend of improved diet quality among high diet cost consumers is the higher intake of alcohol. The mean daily intake of women in the highest cost quartile (9.5 g/d) borders on the upper recommendation in the NNR at 10 g/d. Higher average alcoholic intake among high SES groups has been reported before (43); however, it can be hypothesized that there is a large cultural component to the present result due to the high price of alcoholic beverages in Sweden compared to many other countries.

Another exception is the intake of protein. The mean intake is above (+10%) the recommended maximum intake for the highest cost quartile. This could be interpreted as indicating that the high cost quartile does not in all respect follow a more nutritious diet. Similar findings exist in the literature (5). However, it should be remembered that the variation in protein intake is large within the group and the intake is not significantly larger than 20 g/d in the quartiles (*p=*0.10). Thus, it could not be concluded that a general population of high-cost quartile consumers has an average intake above recommendations at this point.

On the level of individual nutrients, previous studies have found significantly higher intake for consumers with higher energy-adjusted diet cost but differs in respect to which nutrients this applies to (1–3, 5, 7). Diet cost is also expected to differ between groups of different SES and a recent review of differences in individual nutrient intake between groups of different SES points especially to differences in calcium, selenium, copper, and vitamins C (8). Of the four of these nutrients that were included in this study, all but calcium showed significant differences between low and high diet cost consumers.

### Differences to the general population

Dietary habits in the above-average interested and knowledgeable sample studied could be compared to the general population as reported by a recent (2011) large general Swedish study (33). At the individual nutrient level, intake is within 1 standard deviation of the mean of what was reported for the national sample, except for folate, niacin, selenium, and vitamins B12 and C, for which the intake is higher in this study. When comparing average values only, even the lowest cost quartile shows a larger intake of micronutrients than the national sample, which could indicate an effect of interest and knowledge. However, the differences are not statistically significant and larger samples are needed to follow up on this indication.

Due to large variations between individuals in this study, no statistically significant differences with regard to intake of any of the food groups can be identified (Mann–Whitney *U*-tests of differences between medians have *p>*0.16). However, the studied group does on average follow a more exclusive diet with more meat, fish, alcohol, and vegetables and less potatoes, pasta, and bread compared to the national average, despite the fact that students have on average a lower income than the general population. Most striking, however, is the drastic increase in consumption of spreads and fats as compared to the previous study. However, since none of these differences are significant, since it cannot be guaranteed that the categorization of food items is exactly the same, and since the two studies are separated by almost 4 years of time, during which new trends might have evolved, more studies are needed to clarify and study these apparent differences.

### Possible causes for the observed difference

The results thus show a difference in diet quality depending on diet cost even among a sample of above-average interested and knowledgeable consumers. Why does this difference then prevail? Students in Sweden generally represent low-income households. One hypothesis is that dietary choices in low-income groups are limited by budget constraints, leading to poor diet quality (22, 23). Previous studies have suggested that a gradient in budget constraints might translate into an unfavorable gradient in energy density (44), a decreased intake of meat, and an increased intake of, for example cereals (45), similar to what is seen in this study. The higher percentage of older students in the higher cost quartiles also supports this relationship since it is more likely that older students have a higher income or have a partner with higher income. Furthermore, there is a relationship between the cost of obtaining a nutrient and the prevalence of insufficient intake, according to the shadow price analysis.

However, this view has also been challenged by intervention studies showing that diet cost does not necessarily increase when increasing compliance to recommendations (25–27). The relationship between nutritional intake and diet quality is complex and must be understood in relation to palatability and personal preferences (46). Recent studies show that the cost of meeting nutritional recommendations in Sweden is lower than 33–95 SEK/d (24), even when introducing severe palatability constraints. This can be compared to the 71 SEK/d average for the lower diet cost quartile. Moreover, food group preferences (Table 3) in the low diet cost group cannot be explained by budget constraints alone since the foods consumed by the low diet cost quartile are not necessarily efficient in delivering the required nutrients. For example, the lower diet cost quartile has a high consumption of sausage. By dividing the per-weight cost and protein content of various foods, the price of protein obtained from different foods can be calculated. The most commonly consumed sausage provides protein at 0.82 SEK/g, whereas chicken would supply it at 0.35 SEK/g and soybeans at 0.15 SEK/g. Similarly, the average per-unit-mass cost of vegetables and root vegetables is lower than for fruit juices. Thus, the recommended >500 g/d of greens could be supplied at a lower cost by reducing fruit juice and increasing vegetable consumption. This indicates that there is still room for improving nutrition compliance without greatly increasing cost within the low diet cost group.

This study shows that there are differences in diet quality between consumers with high and low energy-adjusted diet cost even when they are all above-averagely interested and knowledgeable in the field of nutrition. However, this does not necessarily imply that it is economically unfeasible for these consumers to significantly improve diet quality without increasing cost. Previous studies have pointed to the importance of finding groups of consumers that are able to consume nutritional diets at low cost, in an attempt to reduce nutritional inequality (6). Although nutritionally knowledgeable consumers do not appear to be that group, the search should continue in future studies.

### Strengths and limitations

The validity and reliability of the results rely on the validity and reliability of the diet recording and the estimated diet costs. The present diet recording study sample is relatively small compared to studies focusing on describing the general population. The risk of using a small sample is a lower reliability and statistical power and thus an increased risk of leaving out differences that would be significant with larger samples. A larger sample would also make it possible to investigate how the above-average interested and knowledgeable sample differs from the national.

A strength of the smaller sample in the present study is the use of a full open diet recording. Many of the previous studies (4, 7) relied on food frequency questionnaires or diet history questionnaires (3, 5), which risks not giving as a detailed view of intake. The respondents had also studied dietary survey methods prior to carrying out the survey and were thus more used to the method than the general self-reporting respondent; on the other hand, respondents were not monitored as would have been optimal. For example, although informed to do so when possible, it was not possible to assure that the respondents used direct weighting of foods. It is well known that estimated weights significantly decrease reliability (47). Furthermore, diet recordings were only conducted for 2 days. The between-days diet variation for an individual is large, and a low number of recording days might result in an overestimation of the across and within quartile variation in Tables 1–3 (47), which could have reduced the number of significant differences between quartiles. The results are also dependent on the quality of the nutritional tables. The overwhelming number of food item compositions was obtained from the official Swedish database in order to reduce this risk.

It is also well known that self-reporting can lead to underreporting; however, it is not expected to influence the general trend of the study since underreporting is more common in less nutritious foods and among individuals with a less nutritious intake (48, 49). Standard exclusion criteria were also used in order to eliminate implausible intake based on energy intake and physical activity. However, since group average PAL values were used for a majority of the respondents, this will somewhat decrease the effectiveness of this, mainly by excessive exclusion of respondents with deviating activity levels.

The diet costs estimate from the online supermarket will not correspond to the prices paid by the respondents; however, no such uniform prices can be obtained since food prices vary over time and between regions. Previous studies have shown that price estimation from supermarket prices has a higher validity than when asking respondents to estimate actual cost (34), and online supermarkets have the advantage of distributing over a wider area than a typical specific supermarket. Furthermore, their prices are more widely available and therefore expected to be more susceptible to market forces, thus describing general price trends well.

The study sample is described as above-average interested and knowledgeable since they were studying university-level nutrition. There is no guarantee that all the respondents had attained the nutritional knowledge specified in the curriculum. However, since diet recording is a demanding task, students that participated tended to be actively participating in the course. Of the 397 respondents, 89% had passed the course as of December 2014. One basic course in nutrition could not make the respondents experts, and due to differences in both pre-existing knowledge and effort spent on the course, differences in knowledge across the sample are expected; however, as a group they are likely to have attained sufficient knowledge corresponding to the more well-informed consumers. The results thus show that the relationship between diet cost and diet quality remains even among a group with a significantly higher minimum level of nutritional knowledge than the population in general.

## Conclusions

The investigated group of nutritionally above-average interested and knowledgeable consumers displayed many of the differences in diet quality across diet cost that have been shown in previous studies on consumers in general: consumers spending more money on food also consumed diets with lower energy density and higher micronutrient density and have a higher energy-adjusted intake of vitamins and minerals that are on average consumed below recommendations. This in turn is caused by a higher intake of vegetables, fruits, berries, and fish among the group spending more money on food. Furthermore, the nutrients consumed below recommendations are often nutrients that are costly to obtain.

The results suggest that a high level of nutritional knowledge does not *per se* eliminate dietary inequality.
